# A pilot clinical phase II trial MemSID: Acute and durable changes of red blood cells of sickle cell disease patients on memantine treatment

**DOI:** 10.1002/jha2.11

**Published:** 2020-05-20

**Authors:** Asya Mahkro, Inga Hegemann, Elena Seiler, Greta Simionato, Viviana Claveria, Nikolay Bogdanov, Clelia Sasselli, Paul Torgerson, Lars Kaestner, Markus G. Manz, Jeroen S. Goede, Max Gassmann, Anna Bogdanova

**Affiliations:** ^1^ Red Blood Cell Research Group, Institute of Veterinary Physiology, Vetsuisse Faculty University of Zurich Zurich Switzerland; ^2^ Zurich Center for Integrative Human Physiology (ZIHP) University of Zurich Zurich Switzerland; ^3^ Department of Medical Hematology University Hospital Zurich Zürich Switzerland; ^4^ Theoretical Medicine and Biosciences Saarland University Homburg Germany; ^5^ Experimental Physics Saarland University Saarbrücken Germany; ^6^ Centre de Biochimie Structurale, CNRS UMR 5048, INSERM UMR 1054 University of Montpellier Montpellier France; ^7^ Division of Medical Oncology and Hematology Cantonal Hospital Winterthur Winterthur Switzerland; ^8^ Section of Epidemiology Vetsuisse Faculty University of Zurich Zurich Switzerland

## Abstract

An increase in abundance and activity of N‐methyl D‐aspartate receptors (NMDAR) was previously reported for red blood cells (RBCs) of sickle cell disease (SCD) patients. Increased Ca^2+^ uptake through the receptor supported dehydration and RBC damage. In a pilot phase IIa‐b clinical trial MemSID, memantine, a blocker of NMDAR, was used for treatment of four patients for 12 months. Two more patients that have enrolled into the study did not finish it. One of them had psychotic event following the involuntary overdose of the drug, whereas the other had vertigo and could not comply to the trial visits schedule. Acute and durable responses of RBCs of SCD patients to daily oral administration of memantine were monitored. Markers of RBC turnover, changes in cell density, and alterations in ion handling and RBC morphology were assessed. Acute transient shifts in intracellular Ca^2+^, volume and density, and reduction in plasma lactate dehydrogenate activity were observed already within the first month of treatment. Durable effects of memantine included (a) decrease in reticulocyte counts, (b) reduction in reticulocyte hemoglobinization, (c) advanced membrane maturation and its stabilization as follows from reduction in the number of NMDAR per cell and reduction in hemolysis, and (iv) rehydration and decrease in K^+^ leakage from patients’ RBC. Memantine therapy resulted in reduction in number of cells with sickle morphology that was sustained at least over 2 months after therapy was stopped indicating an improvement in RBC longevity.

## 1 INTRODUCTION

Sickle cell disease (SCD) is recognized by the World Health Organization as a featured global mortality burden [1]. The cause of this disease is a point mutation in the β globin chain making the deoxygenated form of hemoglobin S (HbS) poorly soluble and prone to form rod‐shape complexes that gives the cells famous “sickle” or “holy leaf” morphology, damages the membrane, and makes RBCs of SCD patients stiff and short‐lived [2, 3]. Deoxygenation, dehydration, and inflammation trigger HbS aggregation, red blood cell (RBC) membrane damage, and ultimately result in production of terminally sickled, highly adherent and fragile cells. This condition is associated with hemolysis, vaso‐occlusive crises (VOCs) and pain episodes, and requires hospitalization, analgesics, and blood transfusions [4, 5]. Preventive approaches include infection prophylaxis and chronic hydroxyurea (HU) that induces production of fetal hemoglobin (HbF) by a mechanism not yet fully understood, possibly involving the nitric oxide (NO)‐driven signaling pathway [2, 6, 7, 8]. Some SCD patients remain refractive to HU treatment, the others may suffer from a number of side effects [9]. Three further drugs, l‐glutamine, Voxelotor, and Crizanlizumab‐tmca, were recently approved for treatment of symptomatic SCD [10]. l‐Glutamine is presumably acts via antioxidant supporting NADH production [11]. Monoclonal anti‐P‐selectin antibody Crizanlizumab targets vaso‐occlusion [12], whereas Voxelotor increases O_2_ affinity of HbS interfering with its aggregation [13]. Several other drugs have been tested, some of which failed, the others being at different stages of testing in clinical trials [10].

An alternative strategy aiming at counteraction of aggregation of HbS was based on targeting dehydration of RBC, reduction in mean corpuscular hemoglobin concentration (MCHC), and thus limiting the interaction between the HbS molecules. The drug of choice, Gardos channel blocker Senicapoc, successfully decreased hemolytic activity but failed to affect the incidence of VOCs [14]. Abnormally high activity of Gardos channels is supported by accelerated Ca^2+^ uptake into RBCs of SCD patients. Apart from activation of Gardos channels, Ca^2+^ overload stimulates NADPH oxidases and promotes proteolytic activity of μ‐calpain [15]. We have identified N‐methyl D‐aspartate receptor (NMDAR) as one of the ion channels involved in abnormally high Ca^2+^ uptake in RBCs of SCD patients [16]. In vitro treatment of patients’ RBCs with the channel blockers, memantine or MK‐801, caused rehydration, reduced the amount of oxidized glutathione, and interfered with sickling [16]. Based on these ex vivo findings, a group of six SCD patents observed at the Clinic of Medical Oncology and Hematology at the University Hospital Zurich were enrolled into a pilot phase II study in which Memantin Mepha was applied to those patients. The primary objective of the clinical study “Safety and tolerability of Memantin Mepha in Sickle Cell Disease Patients (MemSID)” was to assess safety and tolerability of the drug already approved worldwide for treatment of dementia [17, 18] for a group of patients with a different disease. Findings on safety and tolerability of memantine therapy as well as the changes in the quality of life of patients within the MemSID trial are out of the scope of this manuscript and will be summarized elsewhere [19]. We have profited from a unique possibility to assess acute and durable responses of RBCs of SCD patients to the drug‐blocking NMDARs. In order to investigate the impact of memantine on the RBCs form patients suffering from SCD, changes in RBC production rate, hemolytic activity, RBC stability, and RBC sickling were monitored over 12 months of memantine administration.

## 2 MATERIALS AND METHODS

### 2.1 Patients and trial design

The pilot MemSID clinical trial (#NCT02615847 at ClinicalTrial.gov registry) is a phase II, open‐label, single‐center study conducted at the Clinic of Medical Oncology and Hematology at the Zurich University Hospital (USZ) to evaluate the safety and tolerability of memantine for young adult SCD patients. The protocol was approved by the local ethics committee of Canton Zurich (KEK‐ZH 2015‐0297) and the regulatory authority. All participants gave signed informed consent. The study has been conducted in accordance with local ethics committee guidelines and the Declaration of Helsinki. All co‐authors had access to raw data that are available on demand from AB (annab@access.uzh.ch) and AM (makhro@vetphys.uzh.ch).

Six adult patients presenting with symptomatic SCD were included in the study (see Table 1 for further details). Patients P1, P2, P4, and P6 were on HU prior to the trial onset and remained on this medication during the memantine therapy. Patients P3, and P5 were off HU treatment prior and during the trial. A schematic representation of the trial design is shown in Figure S1. Memantine dose was gradually increased (up‐dose phase) from 0 to 20 mg/day over the course of 4 weeks. Maximal dose was maintained for 10 months (treatment phase) and then decreased stepwise during 4 weeks from 20 to 0 mg/day (down‐dose phase). Observations were then continuing for further 8 weeks (follow‐up phase).

**TABLE 1:** Information on the patients enrolled into the MemSID trial

ID	Sex	Age	Origin	HbSS (%)	^*^HbF, % (HU dose, g/day)	Trial course
P1	M	24	Angola	82.6	2.6 (1.5)	Completed
P2	F	48	Kongo	81.5	14.4 (1.5)	Completed
P3	M	30	Afghanistan	78.2	18 (off)	Completed
P4	F	20	Ghana	84.9	8.4 (1.5)	Completed
P5	F	34	Dom. Republic	75	8.8 (off)	Interruption due to psychosis symptoms
P6	F	19	Angola	88	7 (1.0)	Interruption due to vertigo and timing conflict

* HbF values shown are the ones prior to the onset of memantine therapy.

### 2.2 Clinical blood parameters

Laboratory assessments—whole blood count, red cell parameters, and reticulocytes—were measured by an ADVIA 2100 analyzer. Plasma lactate dehydrogenase (LDH) activity was measured using UV spectroscopy. Mean corpuscular hemoglobin concentration (MCHC) was obtained based on spun hematocrit values.

### 2.3 Metabolic activity and K^+^ loss

RBCs were gently spun and washed three times with plasma‐like solution supplemented with 0.1% bovine serum albumin (for details, see Methods section in the Supporting Information). RBCs were resuspended in the same solution to Hb levels of 90‐100 g/L, incubated in a thermoshaker at 37°C and under continuous shaking for 6 h. Every hour extracellular K^+^ concentration was measured, and kinetics of its accumulation plotted against time and normalized to the Hb content.

### 2.4 Flow cytometry for detection of CD71+ and intracellular Ca^2+^

The number of RBCs positive for CD71 (reticulocytes) and intracellular free Ca^2+^ levels was assessed using Gallios Flow Cytometer (Becton Dickenson AG, Allschwil, Switzerland) in 100 000 cells stained with fluorophore‐conjugated antibodies and fluo‐4.

### 2.5 Morphology and morphometry

Two approaches were used to evaluate RBC morphology. Native RBCs were imaged using bright light microscopy within 2‐4 h after blood withdrawal. Along with that RBCs were fixed with 1% glutaraldehyde in a phosphate buffer saline solution within minutes after blood collection. Details of morphometric analysis may be found in Methods section in the Supporting Information and in Figure S3.

### 2.6 RBC density measured using separation on Percoll gradients

RBCs were fractionated into low (L), medium (M), and high (H) density fractions on Percoll density gradient as described elsewhere [20]. Images of the distribution of RBCs within the gradient were taken in front of a homogeneous light source and analyzed using ImageJ software (see Figure S2).

### 2.7 Assessment of the number of NMDARs per cell using the [^3^H]MK‐801 binding assay

The radiolabeled NMDAR antagonist [^3^H]MK‐801 binding assay was used to detect the number of active receptor copies in RBCs forming the M density fraction as described elsewhere [21].

### 2.8 Statistical analysis of the obtained data

All data were analyzed using the R statistical software platform [22]. A linear mixed model using the package lme4 [23] was used to examine the association of each clinical or laboratory variable with trial phase (pretreatment phase, up‐dose, treatment, down‐dose, and follow‐up). Each patient was used as a random effect in the model implicating repeated measure statistics. Examination of the residuals indicated that a Gaussian model with mixed effects was a reasonable statistical model for this analysis.

## 3 RESULTS

### 3.1 Acute effects of memantine treatment on RBCs

**FIGURE 1:**
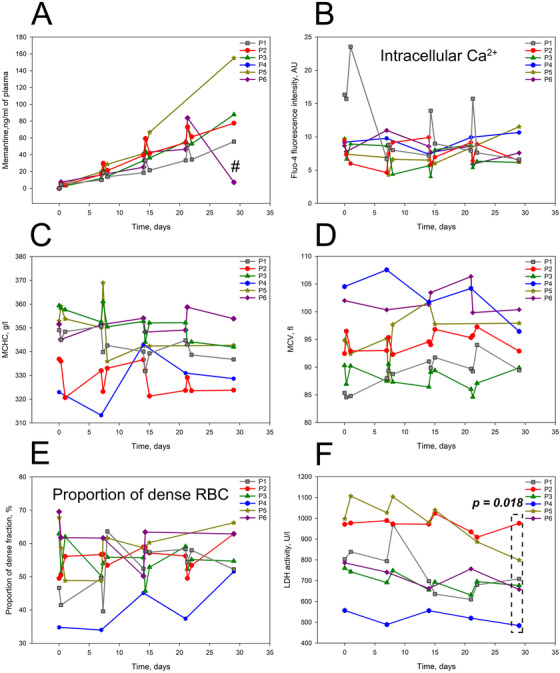
Acute effects of memantine therapy. Plasma memantine levels for SCD patients 1‐6 during the up‐dose phase. # stands for 1 week after the memantine therapy was discontinued (A). Alterations in the intracellular‐free Ca^2+^ measured as fluo‐4 fluorescence intensity (B), MCHC (C), MCV and (D) in RBCs of SCD patients 1‐6 during the up‐dose phase. ECell abundance in high density fraction (E). Plasma lactate dehydrogenase (LDH) activity in patients 1‐6 during the up‐dose phase (F). LDH activity values showed a significant decline by the end of up‐dose period (paired *t*‐test was applied comparing the last to the first value)

During the first 4 weeks of the trial, patients were administered escalating doses of memantine and blood samples were taken before, 6, and 24 h after the drug administration for patients P1‐P3, P5, and P6, whereas patient P4 was only observed weekly during the up‐dosing phase. Plasma memantine concentration was increasing with the dose, but its pharmacokinetics varied from patient to patient (Figure 1A). During the trial, P5 exposed oneself to an involuntary overdose resulting in development of psychosis, which did not allow this patient to continue the treatment. Patient P6 reported vertigo and had difficulties to coply to the trials schedule, and dropped out shortly after the up‐dose phase. Patients 1‐4 successfully completed the 12 months of memantine therapy and were observed for 2 months after the drug administration was stopped (follow‐up phase).

Increment in the memantine dose triggered fluctuations in the intracellular free Ca^2+^ measured as fluorescence intensity of Fluo‐4 (Figure 1B). These changes were associated with a transient shifts in MCHC, MCV, and abundance of dense cells (Figure 1C‐E). Oscillatory changes in LDH activity during the up‐dosing phase were associated with decrease in this hemolytic marker by the end of the first month of memantine therapy (Figure 1F).

**FIGURE 2:**
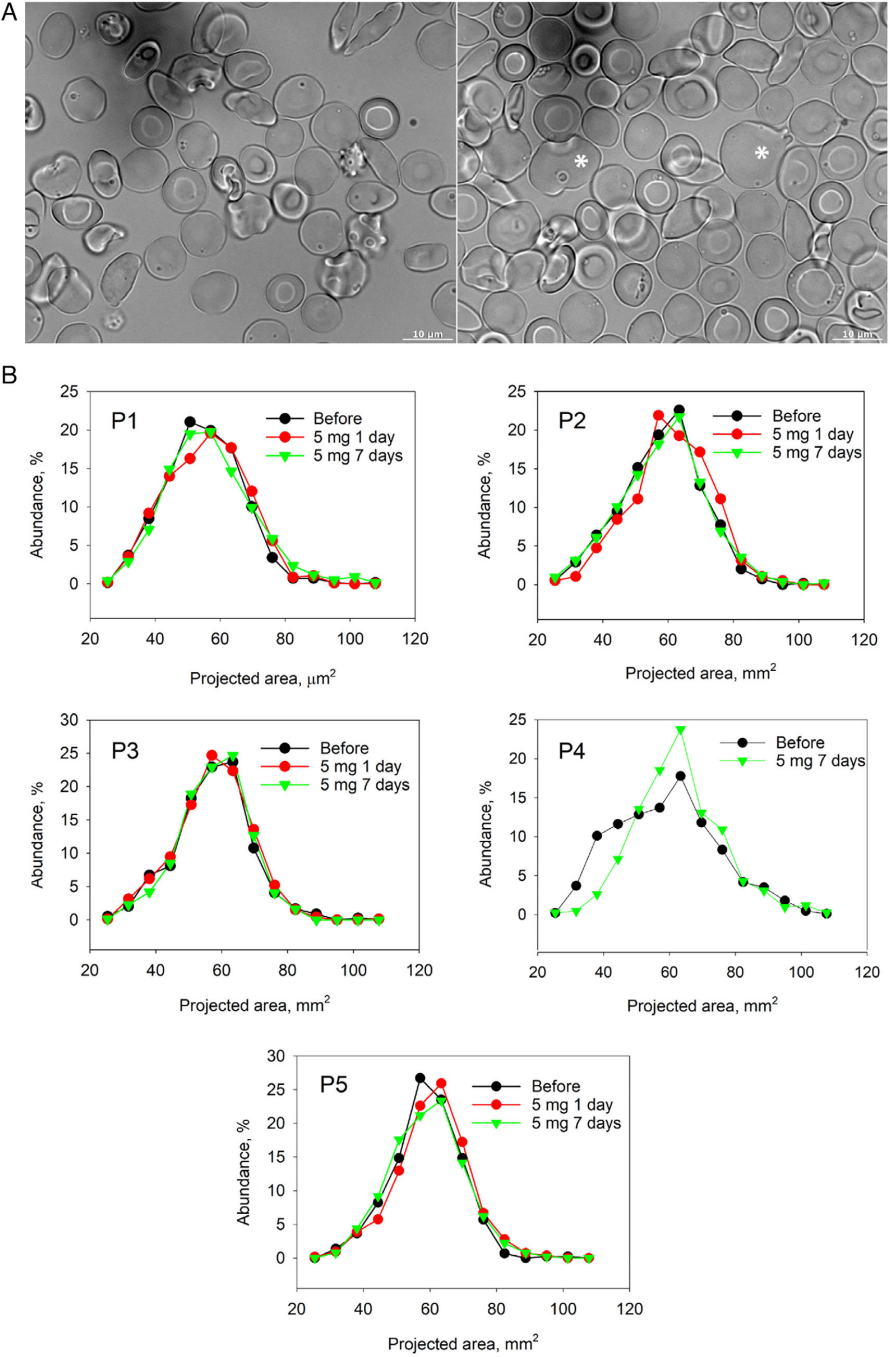
Changes in RBC morphology in response to memantine treatment during the up‐dosing phase. Comparison of RBC morphology of patient P1 before the onset of treatment (left panel) and at the up‐dose phase (right panel) (A). Abnormally large cells are highlighted with the star. Distribution of the projected areas of cells at the onset of the study, during the first day of treatment with 5 mg memantine and after a week of administration of 5 mg memantine a day (B).

Treatment with memantine doses of 5‐10 mg/day was associated with a transient appearance of unusually large RBCs in all patients (Figure 2A). This effect was transient and limited to the up‐dosing phase in all but P4 patient, for whom these large RBCs remained in the circulation during the treatment phase as well (Figure 2B).

### 3.2 Durable effects of memantine therapy

Durable alterations in RBC properties, including RBC indices, developing over the 10 months of treatment of patients with 20 mg memantine per day are summarized in Table 2. Some of these changes persisted after the medication was stopped; some features were restored during the down‐dose and follow‐up phases.

**TABLE 2:** Changes in RBC indices and parameters associated with their turnover and volume regulation in response to memantine therapy

	P1		P2		P3		P4	
	Start	End	Start	End	Start	End	Start	End
Retic. Hb	27.5	24.5^**^	27.9	26.2^*^	–	25.4	31.0	28.6^*^
CD71+ retic. (%)	58.1	66.0	43.2	50.1	35.2	43.8	44.5	46.8
K^+^ leak, µmole/(gHbxh)	228.8	93.0^***^	222.2	145.1^***^	155.4	98.2^**^	251.4	192.1
MCHC (g/L)	343.0	321.7^***^	329.1	318.5^**^	352.7	350.1	327.7	334.6
% Hyperchrome cells	6.46	3.27^***^	2.28	2.27	8.4	5.8^***^	2.0	2.0
% of cells in dense fraction	36.7	22.1^**^	19.0	15.4^***^	39.1	26.6^***^	13.9	11.3
MCV (fL)	88.6	91.2	94.6	92.9	88.7	86.9	102.2	98.9
MCH (g/L)	29.5	28.5^*^	30.4	29.9	31.0	29.5^***^	33.1	31.2^***^
Macrocytes (%)	5.0	3.8	5.1	4.8	3.7	1.6^***^	11.3	8.6
Microcytes (%)	5.6	5.3	2.3	2.9^*^	2.6	4.9^***^	1.4	2.3^***^

*Note*. Average of the values from the start of the MemSID trial to the end of the up‐dosing phase (base) are compared to the average from the last 3 months of treatments (20 mg/day) to the end of the down‐dose phase (end). Stars denote significance (^*^ *P* < .05; ^**^ *P* < .01, ^***^ *P* < .001) between the “base” and the “end” datasets for the individual patients (Student's *t*‐test or Mann‐Whitney Rank Sum test depending on the outcome of normality test).

Abbreviations: Retic. Hb, intracellular hemoglobin in reticulocytes; CD71+retic, immature reticulocytes positive for CD71; MCV, mean corpuscular volume; MCH, mean corpuscular hemoglobin; MCHC, mean corpuscular hemoglobin concentration.

#### 3.2.1 Reticulocyte count and changes in hemoglobin types and levels

**FIGURE 3:**
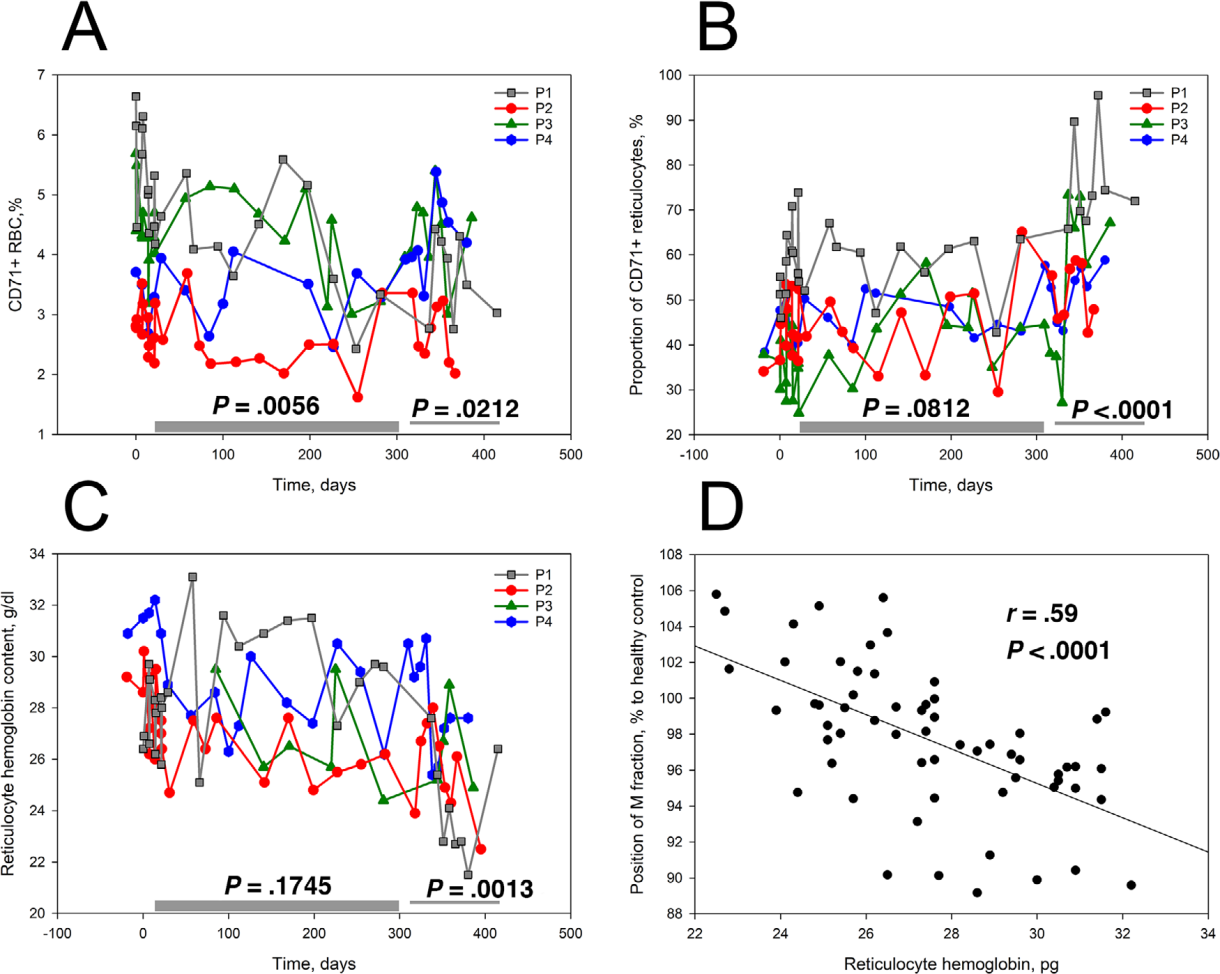
Impact of durable memantine therapy on reticulocytes in patients 1‐4. Abundance of CD71+ reticulocytes (A), reticulocytes’ maturation state (CD71+/RNA+ cells; (B)) and their hemoglobinization (C) in SCD patients 1‐4 during the treatment, down‐dosing and follow‐up phases. D, Density of mature RBCs as a function of hemoglobin content of reticulocytes for cell of SCD patients on memantine trial. Bars indicate the treatment phase and the down‐dose and follow‐up periods. Significance of changes between the trial phases was assessed using a linear mixed model in which phases were chosen as variables and patients used as a random effect. The *P*‐values are shown when the treatment and down‐dose/follow‐up phases were compared with the pretreatment phase

A sustainable decline in reticulocyte counts during the treatment phase was observed (Figure 3A). At the same time, the proportion of early stage reticulocytes positive for CD71 and RNA in total reticulocyte population was slowly growing during the treatment phase and was particularly high (62.9 ± 11.2%) during the follow‐up phase (Figure 3B). Memantine therapy also caused a decrease in reticulocyte hemoglobinization that persisted in a follow‐up phase (Figure 3C). Decrease in reticulocyte hemoglobin content was associated with reduction in density of mature RBCs (M fraction within Percoll gradient, Figure S2) of patients (Figure 3D).

**FIGURE 4:**
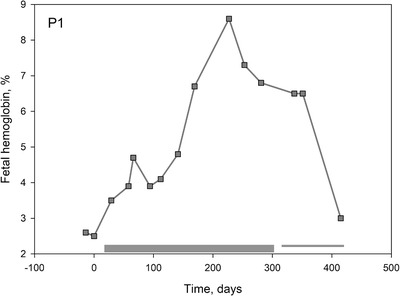
Time‐course of changes in HbF abundance for patient P1 during the trial

Patient P1 was chronically receiving HU but was unable to increase fetal hemoglobin (HbF) levels above 2% prior to the trial (Table 1), whereas for the other patients this value ranged between 9% and 18%. For P1, memantine therapy resulted in an increase in HbF to 8% (Figure 4). This patient benefited so much from the new treatment; he was restored on memantine therapy after the MemSID trial was over.

#### 3.2.2 Changes in RBC hydration and density

**FIGURE 5:**
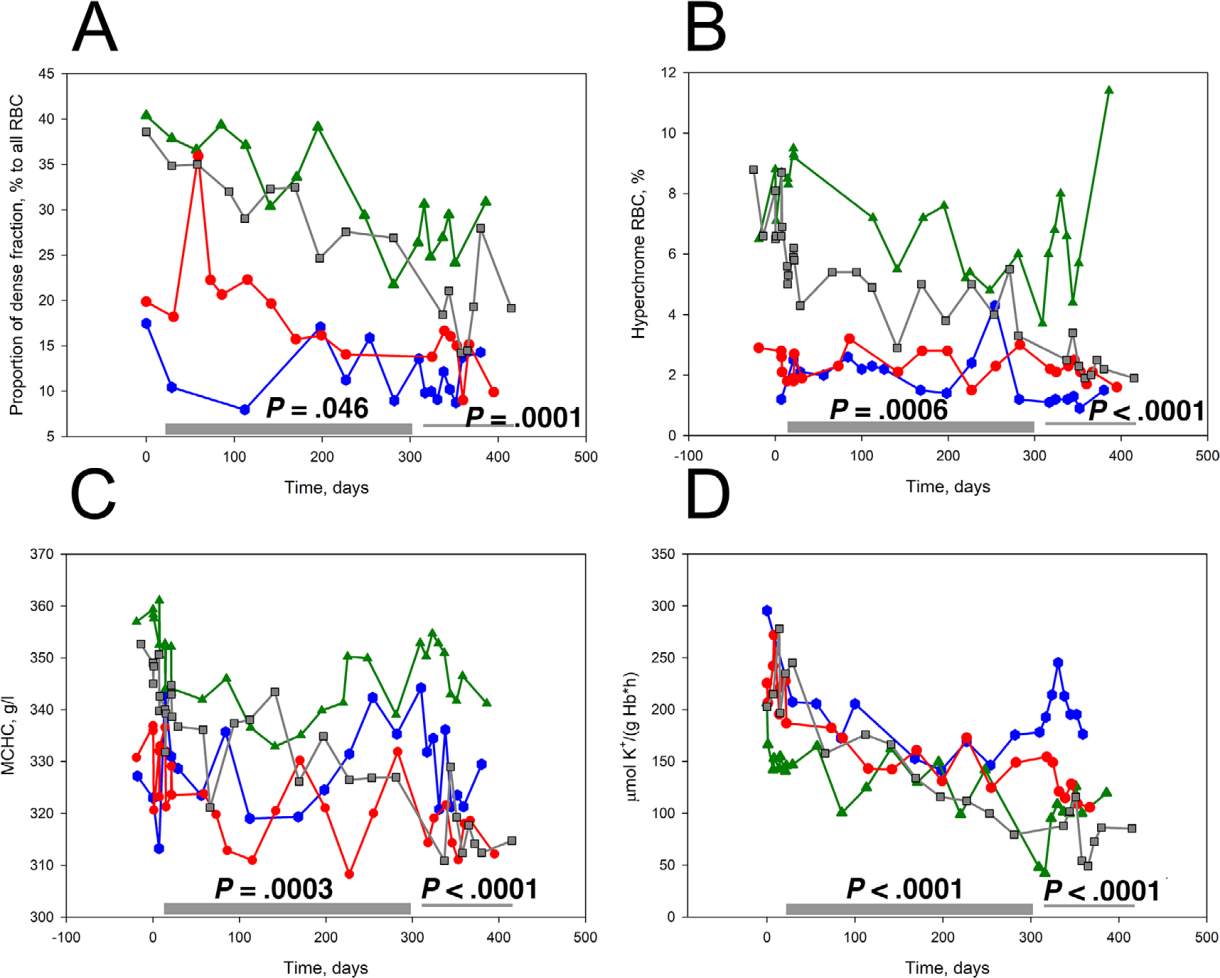
Effect of memantine treatment on RBC hydration state and membrane maturation in patients 1‐4. Impact of durable memantine therapy on the abundance of dense cells (A) and hyperchrome cells (B), MCHC (C), and passive K^+^ leakage from RBCs (D). Significance of changes between the trial phases was assessed using a linear mixed model in which phases were chosen as variables and patients used as a random effect. The *P*‐values are shown when the treatment and down‐dose/follow‐up phases were compared with the pretreatment phase. Color‐coding for the P1‐P4 is similar to that in Fig. 3

**FIGURE 6:**
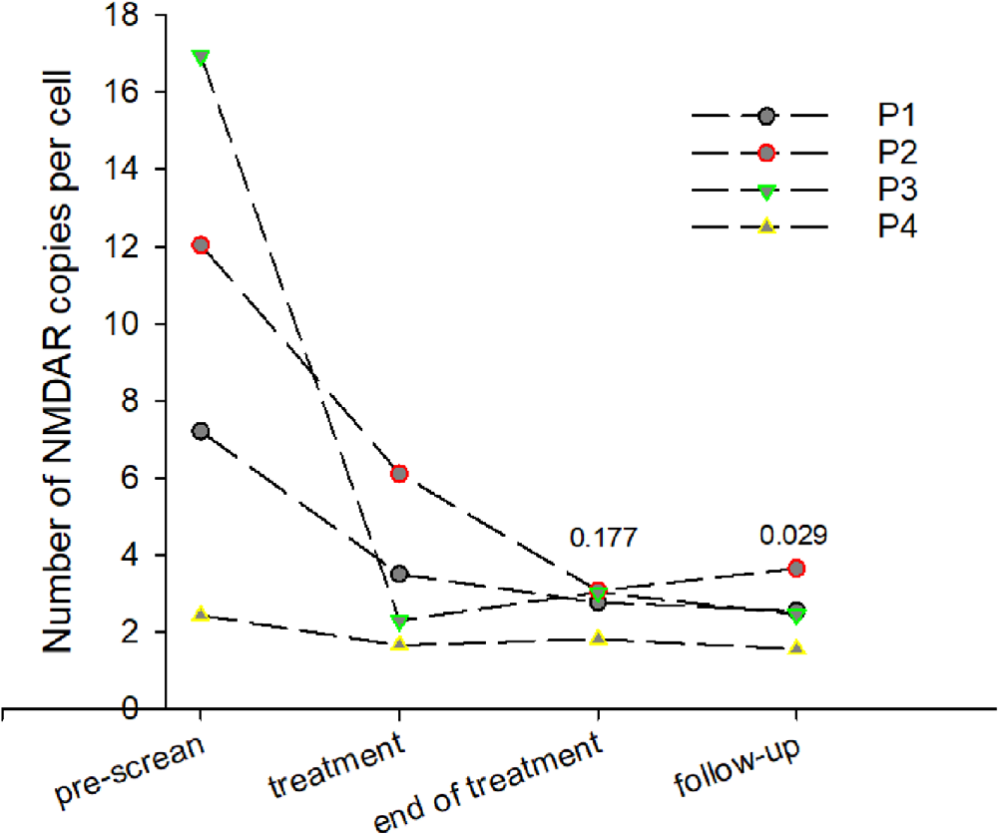
Changes in abundance of NMDARs in mature RBCs as a result of memantine treatment. One‐way repeated measures ANOVA on ranks with was applied to estimate the difference in receptor abundance over the treatment phases (compared to the pretreatment phase). Numbers above the graphs are the p values

Several parameters revealed alterations in RBC hydration state in the course of durable memantine administration. Reduction in cells forming heavy fraction on Percoll density gradient along with the number of hyperchrome cells was preserved during treatment, down‐dose, and follow‐up phases (Figures 5A and 5B). These changes were in line with the decrease in MCHC levels (Figure 5C). Along with persisting reduction in passive K^+^ leak from the cells (Figure 5D), reduction in number of NMDARs per cell was observed in response to memantine treatment (Figure 6).

#### 3.2.3 Changes in RBC morphology

Effects of memantine treatment on RBCs morphology and intracellular Ca^2+^ distribution were monitored microscopically. Abundance of terminally sickled cells was assessed for both glutaraldehyde‐fixed RBCs and native cells of SCD patients. Subsequently, the outcome of detection of terminally sickled cells for these two types of samples was compared to avoid the artefacts of fixation (which was done immediately after blood withdrawal) and the possible reversal from sickle to normal morphology that could occur with time before the native cells were imaged. Eccentricity and solidity were used as a quantitative marker for detection of sickling (Figures S3B and S3C).

**FIGURE 7:**
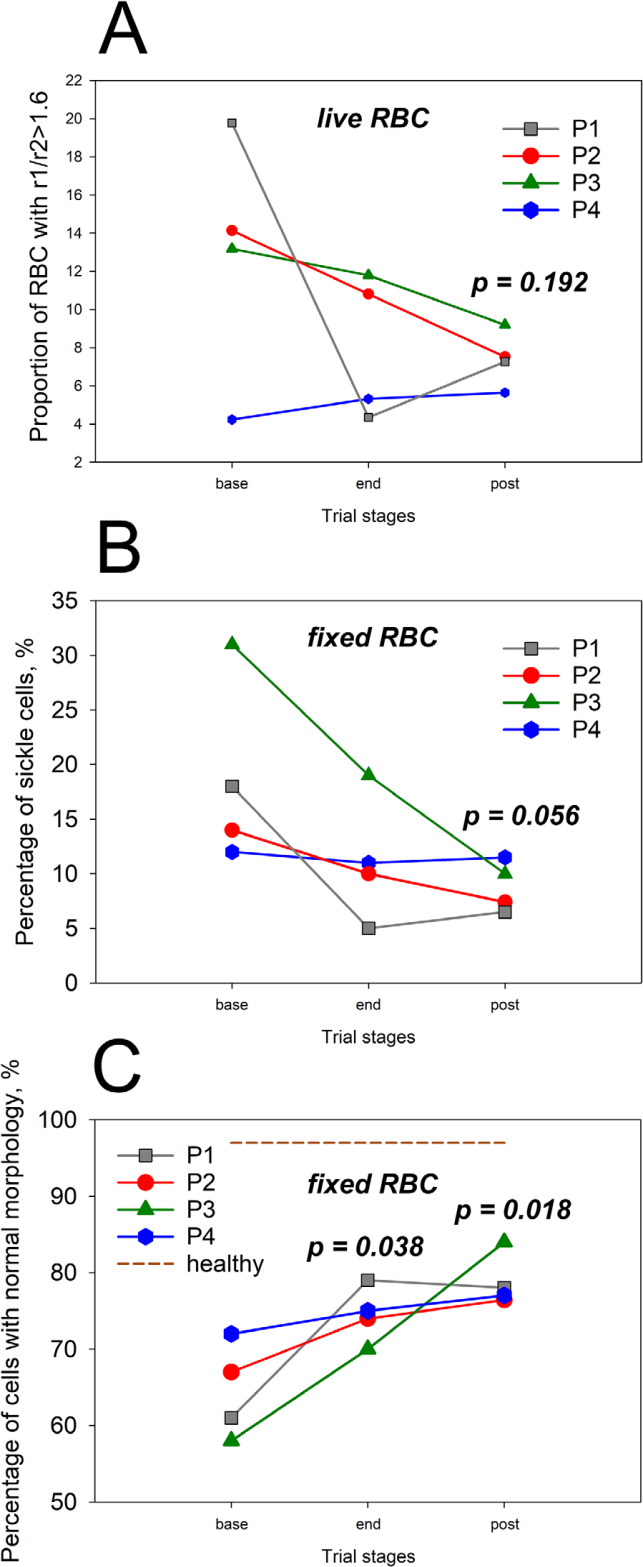
Morphological changes in RBCs upon memantine therapy in SCD patients 1‐4. Percentage of elongated cells (shortest to longest diameters R1/R2 > 1.6) observed for native cells using bright‐field microscopy (A). Percentage of fixed RBCs with sickled morphology (B). Abundance of fixed cells with normal morphology (C)

The number of cells with sickle morphology declined for both native (Figure 7A) and fixed (Figure 7B) RBC indicating that the observed sickling was indeed irreversible. At the same time, the abundance of cells with “normal” morphology increased for all patients by the end of the treatment period (Figures 7C and S3).

Stabilized morphology was preserved after cessation of the memantine therapy. Representative images of RBC shapes of three healthy human subjects and a SCD patients P1‐P4 at screening (base) after the treatment phase (end) and by the end of a follow‐up (post) phase are shown in Figure 8A. The complementary fluorescent images were taken to evaluate intracellular Ca^2+^ distribution in RBCs of healthy people and SCD patients. The cells of healthy donors contained exclusively nanovesicles filled with Ca^2+^, whereas RBCs of patients before the onset of treatment were filled with multiple larger Ca^2+^‐storing microvesicles (Figure 8A). The abundance of these compartments was reduced by the end of treatment period.

FIGURE 8Calcium sequestration and the impact of Ca^2+^‐targeting memantine therapy on RBCs of SCD patients. (A) RBC morphology and the intracellular Ca^2+^ distribution in RBC of healthy donors (control1‐control3) and for patients P1‐P4 before the onset of treatment (base), by the end of treatment (end) and by the end of follow‐up phase (post). Arrows highlight nanovesicles filled with Ca^2+^ in cells of healthy patients. (B) Summary on the effect of memantine on RBC of SCD patients. Memantine therapy resulted in reduction in hemoglobin content in reticulocytes and RBCs, a decrease in the number of NMDARs per cell and improved hydration that was caused by a decline in K^+^ leak. As a result, irreversible sickling was avoided, and the longevity of RBCs improved

**Figure:**
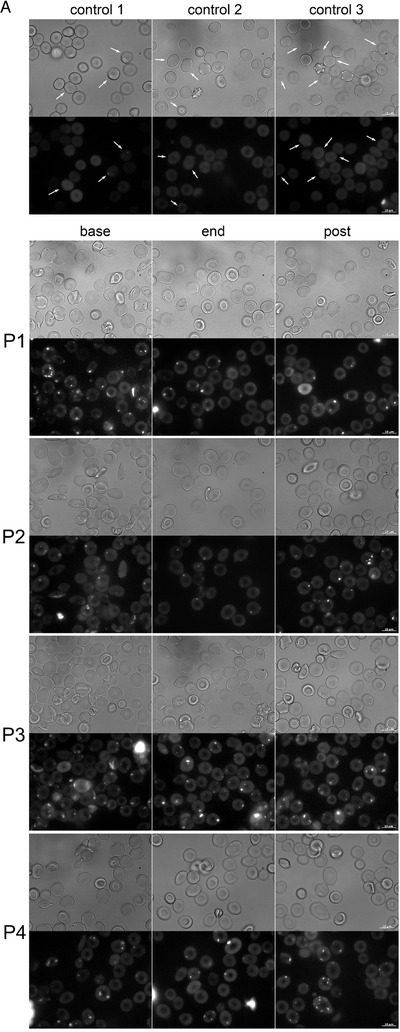

**Figure:**
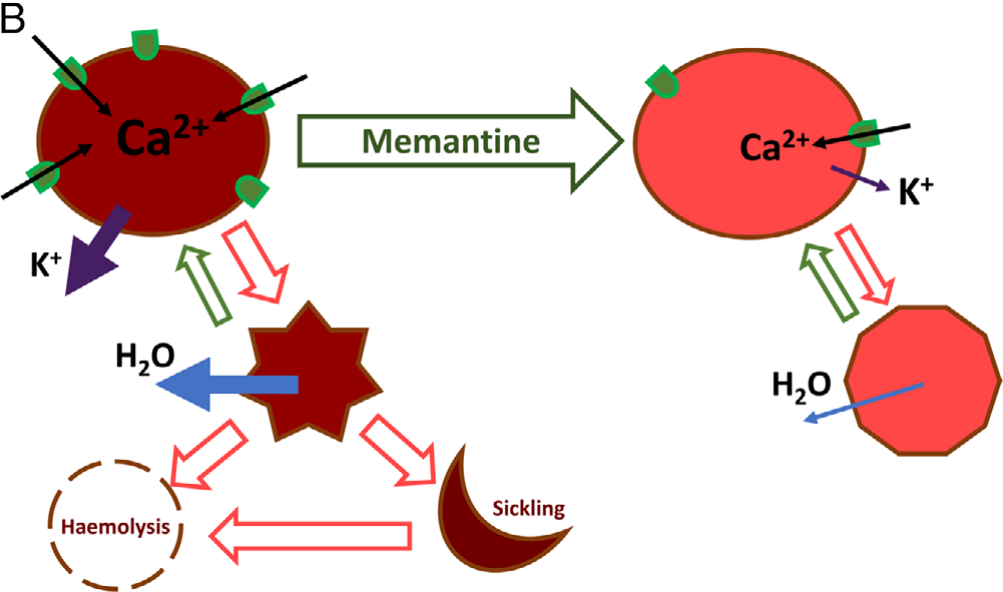

As presence of NMDARs was shown for white blood cells (WBCs) [24, 25, 26, 27, 28] and platelets [29] along with RBCs, we have assessed the possible effects of memantine on WBC and platelet counts. Data on the lack of impact on the WBC and platelet counts are presented in the Table S1.

## 4 DISCUSSION

We have observed several acute and durable effects of memantine therapy on RBC properties of SCD patients. Some of these changes most likely reflect responses of erythroid precursor cells in the bone marrow to the NMDAR antagonist. The others are associated with the action of the drug on the circulating RBCs. The observed changes are schematically summarized in Figure 8B. First responses were triggered at plasma memantine concentrations within nanomolar range, much lower than those (10‐50 μM) we have applied when monitoring the NMDAR activity in RBC suspensions [16, 30]. In vivo, the first responses were observed when plasma memantine concentrations reached on average 22 nM (4.1 ± 0.8 ng/mL) for 5 mg/day dose, and persisted at ∼378 nM (65.6 ± 16.2 ng/mL) for 20 mg/day dose. These findings suggest that NMDAR channel opening probability increased in RBCs that were exposed to both agonists (glutamate and glycine) and shear stress. These conditions most likely facilitated memantine interaction with its binding site within the channel pore. Earlier on shear stress was found to activate NMDARs in astrocytes and cardiomyocytes [31, 32].

Influx of Ca^2+^ through the NMDAR is targeted by memantine directly triggering multiple calcium‐dependent signaling events. In mature RBCs Gardos channel, inositol triphosphatase and protein kinase C, NADPH oxidases, and calpain are among the downstream targets of Ca^2+^ signaling [15]. Less is known about the Ca^2+^‐driven signaling cascades in erythroid precursor cells [33, 34]. Monitoring of acute response of free Ca^2+^ levels as well as Ca^2+^‐dependent parameters, such as RBC volume and density to memantine therapy, revealed their acute transient changes (Figure 1B‐E). Each of these parameters is known to have a feedback‐controlled mechanism. Ca^2+^ uptake is counteracted by channel deactivation and activation of Ca^2+^ pumps[35]; loss of K^+^ and water may be transient and activates regulatory volume increase mechanisms [36]. Hence, instability may reflect the impact of inhibition of NMDARs on Ca^2+^ handling and Ca^2+^‐dependent parameters. LDH activity in plasma is a reliable marker of hemolysis in SCD patients [37]. It shows consistent decline within the first month of treatment (Figure 1F).

Treatment with memantine resulted in production of cells with normal morphology and reduction in abundance of abnormal or terminally sickle cells (Figure 7). Persistence of these changes as well as of effective NMDAR shedding and low plasma LDH levels during the follow‐up phase (Figure 6) indicated that the cells produced during memantine therapy were longer lived.

Causes for improved longevity included reprogramming of precursor cells in the bone marrow as well as the stabilization of the circulating RBCs. Durable exposure to memantine decreased reticulocytes’ hemoglobin content (Figures 3A and 3C) and, in one case, enriched with the circulating cells with HbF (Figure 4). Interruption of treatment triggered production of immature reticulocytes (Figure 3B). Calcium‐induced regulation of erythroid differentiation has been reported to occur via calcineurin‐sensitive and calcineurin‐independent Epo‐inducible pathways [38] that involves and cAMP responsive element binding protein and nuclear factor of activated T cells (NFATc2) transcription factors [38, 39, 40]. Recently, participation of Ca^2+^‐permeable channel PIEZO1 in regulation of globin gene expression via NFAT‐driven signaling cascade was reported in human CD34+‐precursors [41]. Possible involvement of this pathway in the changes in hemoglobinization, HbF expression in reticulocytes, and possible reduction in proliferative activity of erythroid precursors of SCD patients upon memantine treatment remains to be investigated.

Along with these changes in properties of erythroid cells, durable reduction in irreversible sickling was observed in response to memantine administration along with shedding from excessive NMDARs (Figure 6). Furthermore, treatment with memantine caused a decline in K^+^ leak and dehydration (Figure 5D) of mature RBCs of SCD patients on memantine treatment. These findings are in line with our former ex vivo observations on the effect of NMDAR inhibition on RBC hydration [16, 30]. In India, zinc sulfate or acetate was provided to SCD patients for replenishment of depleted zinc stores [42, 43, 44]. This therapeutic approach resulted in prevention of dehydration of RBCs. Zn^2+^ supplementation reduced the inflammation and resulted in decreased numbers of sickle cells [44, 45]. One of the targets of Zn^2+^ could be NMDARs that are blocked by divalent cations in neurons, in cell cultures [46, 47] and in cardiac muscle [31]. Myocardial NMDARs resemble the erythroid receptors in their subunit composition [31]. The anti‐inflammatory properties of Zn^2+^ may also be explained by the same mechanism [48].

Targeting dehydration alone by blocking Gardos channel with Senicapoc in SCD patients was proved to be insufficient to decrease the incidence of pain episodes despite very promising amelioration in hemolytic activity [14]. The reason of this paradoxical outcome is not clear till now. It was suggested that an increase in hemoglobin and hematocrit was associated with pathological upregulation of blood viscosity, promoting VOC and pain. In our study, memantine treatment did not cause an increase in number of RBCs (data not shown) and worked to improve hydration state and RBC stability synergistically with HU in few patients participating in MemSID trial. The currently running MEMAGEN trial (#NCT03247218) will provide more insights into the clinical potential of memantine as a supportive drug for patients with symptomatic SCD. Furthermore, extensive trials will be needed to evaluate the impact of memantine therapy on VOCs.

## AUTHOR CONTRIBUTIONS

AB and AM designed the biological part of the study, whereas JG, IH, and MM designed the clinical trial protocol and JG and IH performed the clinical trial. MM and CS were managing the clinical trial. AM, ES, AB, GS, LK, VG, and NB performed the measurements and MG discussed the data. AM, PT, and AB analyzed the data. AB, AM, PT, and MG wrote and critically reviewed the manuscript. All co‐authors read the final manuscript and expressed consent on the content.

## CONFLICT OF INTEREST

The authors declare no conflict of interest. The University of Zurich holds the patent to use memantine against sickle cell disease.

## Supporting information

Supporting Information
