# Supplementary material for: A pilot clinical phase II trial MemSID: Acute and durable changes of red blood cells of sickle cell disease patients on memantine treatment
Source: EJHaem. 2020 May 20;1(1):23–34. doi: 10.1002/jha2.11 (PMC9175962; doi:10.1002/jha2.11)

## Extended materials and methods

*Patients and trial design*

The Clinical Trial to Study the Safety and Tolerability of Memantin Mepha® in Sickle Cell Disease Patients (MemSID, #NCT02615847 at ClinicalTrial.gov registry) is a phase II, open-label, single center study conducted at the Department of Hematology at the Zurich University Hospital (USZ) to evaluate the safety and tolerability of memantine for young adult SCD patients. The protocol was approved by the local ethics committee of the Canton Zurich (KEK-ZH 2015-0297) and the regulatory authority. All participants gave signed informed consent. The study has been conducted in accordance to the local ethic committee guidelines and Declaration of Helsinki. Six adult patients presented with HbSS sickle cell disease (see Table 1). Schematic representation of the trial phases is shown in suppl Fig 1.

The memantine dose was gradually increasing (phases I) and decreasing (phase III) from 0 to 20 to 0 g/day. During 10 months of phase II the drug dose was maintained at 20 mg/day.

*Clinical blood parameters*

Laboratory assessments: Whole blood count, red cell parameters (MCV, hyperchrome RBC) and RNA/DNA bearing reticulocytes were measured by an ADVIA® 2100 analyzer. Plasma lactate dehydrogenase activity (LDH), was measured using UV spectroscopy. Mean corpuscular haemoglobin concentration (MCHC) was detected from hematocrit measurement using a capillary assay. HbS and HbF were monitored by HPLC. Quantitation of plasma memantine concentration was performed using a combination of HPLC and MS/MS mass spectrometry (Noetzli, *et al* 2012, Noetzli, *et al* 2011).

*Metabolic activity and $K^+$ loss*

RBCs were gently spun down and washed three times with plasma-like solution containing (in mM): 140 NaCl, 4 KCl, 0.75 $MgSO_4$, 0.015 $ZnCl_2$, 10 glucose, 0.2 Glycine, 0.2 Na-Glutamate, 0.1 Arginine, 0.6 Glutamine, 20 HEPES-imidazole buffer (pH 7.4 at room temperature) supplemented with 0.1 % bovine

serum albumin (BSA). RBCs were re-suspended in the same solution to Hb levels of 90-100 g/l, incubated in a thermoshaker at 37°C and under continuous shaking for 6 hours. Each hour extracellular $K^+$ concentration was measured, and kinetics of its accumulation plotted against time and normailsed per Hb content.

*Flow cytometry for detection of CD71+ RBC and intracellular free $Ca^{2+}$ content*

The number of RBCs positive for CD71 (reticulocytes) was assessed using Gallios Flow Cytometer (Becton Dickenson AG, Allschwil, Switzerland) for 100 000 cells at medium flow rate. Mouse anti-human clone IgG1, kappa, antibodies against CD71 conjugated with APC (eBiosciences/Affimiterix cat. #17-0719-42, 1:500 dilution) were used. Data analysis was performed using Kaluza Analysis software (Beckman Coulter Life Sciences, Indianapolis, IN USA). Free $Ca^{2+}$ content was estimated after 2h on incubation with 1 µM fluo-4AM (0.1% Hct, RT).

*RBC density measured using separation on Percoll gradients*

RBCs were fractionated into low-, medium- and high-density fractions on Percoll density gradient as described elsewhere[22]. One ml of whole blood was layered on top of 13 ml of the 90% isotonic Percoll solution and centrifuged at 48 000×g at 34-36◦C for 15 min to achieve separation of cells into fraction of low (L), medium (M) and high (H) densities. Image of the distribution of RBCs within the gradient was taken against the homogeneous light source and analyzed using ImageJ software (see Suppl Fig 2). In addition, cells forming L, M and H fraction were then collected and the number of cells within the fractions in % was calculated using capillary hematocrit measurements for whole blood and for reach fraction.

*Assessment of the number of NMDARs per cell using [$^3$H]MK-801 binding assay*

The radiolabeled NMDA receptor antagonist ([$^3$H]MK-801) binding assay was used to detect the number of receptor copies in RBCs forming M density fraction as described elsewhere [25]. Briefly, RBCs were washed with plasma like solution and resuspended to a hematocrit of 40-50%. 5 µl of [$^3$H]MK-801 (1 mCi/ml 27.5 Ci/mM, Perkin Elmer) were added to 1 ml suspension in the presence or absence of 500-fold dose of non-labeled MK-801. Incubated with [$^3$H]MK-801 at room temperature lasted 1 h. Thereafter RBCs were separated into L, M and H fractions as described above. Cells forming the M fraction were harvested and washed from Percoll and from non-bound radiolabeled antagonist three times with 10 ml of plasma-like solution and resuspended in the same solution to 1 ml. Hematocrit was estimated in suspensions and the cells lysed. Membranes of RBCs were dissolved in scintillation fluid and radioactivity of tritium was detected using Tri Carb

4910TR 110 V Liquid Scintillation Counter Scintillation Counter (Perkin Elmer Inc, Waltham, Massachusetts, USA).

The amount of [$^3$H]MK-801 bound per cell was calculated considering the average volume of a single cell of 90fl and the amount trapped water in hematocrit capillary of 5%. Non-specific binding obtained in a parallel sample where [$^3$H]MK-801 was present along with 500-fold excess of non-labeled antagonist was subtracted from the obtained value.

*Confocal imaging methods and analysis*

a) Sample preparation

5 µl of fresh blood were fixed in 1 ml of 1% glutaraldehyde in phosphate buffer saline (PBS) solution (Gibco Life Technologies, Grand Island, USA) and stored at room temperature. All the samples collected by the end of the treatment were then washed three times in PBS by centrifuging cells for 3 min at 10,000 g (Micro Centrifuge 5415 C) between each wash. Afterwards, cells were resuspended in PBS and labeled with 2.5 µg/ml of CellMask$^{TM}$ Deep Red plasma membrane stain (Molecular Probes Life Technologies) for 15 min, then washed three times as described before.

b) Confocal imaging

Labeled cells were placed on glass slides for imaging on top of a 60X objective (CFI Plan Apochromat Lambda 60X Oil, NA=1.4) of an inverted microscope (Nikon Eclipse Ti). A solid-state laser ($\lambda$ = 647 nm, Nikon LU-NV Laser Unit) was used as a light source for imaging. Z-stack scanning was realized with 300 nm piezo stepper. Scanning from top to bottom for a 20 µm z-range. Confocal image generation was performed with a spinning disk based confocal head (CSU-W1, Yokogawa Electric Corporation). Image sequences were acquired with a digital camera (Orca-Flash 4.0, Hamamatsu Photonics, Hamamatsu City, Japan). Representative examples of cell images from the time point before the memantine treatment, by the end of treatment and after the follow-up period (post) are shown on the Supplementary Fig 3A.

c) Image processing

A semi-automatic Matlab R2017b (MathWorks) routine was used to enable the detection and extraction of geometrical characteristics of individual sRBCs. From the stack of images obtained by confocal microscopy, the maximum pixel intensity value was identified for each $(x,y)$ position and projected at $z = 0$ in order to obtain 2D images with homogeneous background and cell border, easily detectable compared to bright field images, where out of plane cell borders are difficult to identify. 2D projected images were then binarized by setting in first place a threshold on the image pixel

intensity value followed by a typical binarization routine. The identified objects (sRBCs) were set to pixel value 1 (white) and the background to pixel value 0 (black). The coordinates of cell borders were defined as the positions of the interface between the objects and the background. The number of pixels inside the cell border was defined as the cell projected area, $A$. From the covariance matrix obtained by a fitting of a multivariate normal distribution to the cell border coordinates, the major and the minor axis, $a$ and $b$ respectively, of an ellipse that fits to the cell contour and its eccentricity $\varepsilon$ were obtained. Eccentricity is defined as $\varepsilon = \sqrt{1-(a/b)^2}$, where $\varepsilon = 0$ represents a perfect circle and $\varepsilon = 1$ a line and its value is used as a measure of cell elongation. The ratio of the cell projected area, $A$, and the area of the smallest polygon that contains the binarized cell, $A_p$, is defined as solidity, $s$, where $s = A/A_p$. Solidity is a measure of the roughness of the cell (or how "spiky" the cell is). $s = 1$ indicates a perfect smooth surface while cells with $s < 1$ has a degree of roughness whose intensity increases when $s$ decreases. The applicability of both parameters was tested on artificially deoxygenated blood (0.1% $O_2$, 5% $CO_2$, 94,9% $N_2$, 37°C, $CO_2$-buffered plasma-like medium, 8 h) of the same SCD patients. Examples of RBC with reduced solidity and increased eccentricity as a result of HbF crystals formation are shown on the Supplementary Fig 3B.

Once area, eccentricity and solidity values from all cells of a sample were obtained, small objects, overlapped and highly drifted cells were filtered out considering a maximum and minimum projected area accepted values.

d) Manual RBC classification

Detected cells were subjected to a manual and individual scrutiny for their classification and to filter out remaining non-applicable detections. The classification was based on three groups: 1) "non-sickle" cells, that includes discocytes, echinocytes and stomatocytes (see Supplementary Figure 4); 2) "sickle", considered as so according to Corbett (Corbett*, et al* 1995) description of cells which bearer single or multicentral HbF crystalls; 3) "others", to include additional shapes (e.g. teardrop cells), and deformed cells without a clear typical shape observed in sickle cell disease. The classification procedure was repeated tree times with an error of 2%. Eccentricity was considered as a reference value to distinguish discocytes from ellyptocytes: cells with $\varepsilon \geq 0.7$ were classified as ellyptocytes and therefore included in the group "others". Changes in the prevalences of those shape groups after the memantine treatment are show on the Supplementary Figure 3C.

**Supplementary table 1**

Effect of memantine therapy on the white blood cell counts in SCD patients

| | P1 | | P2 | | P3 | | P4 | |
|---|---|---|---|---|---|---|---|---|
| | base | end | base | end | base | end | base | end |
| Leucocytes, G/l | 9.61 | 8.56 | 6.16 | 6.11 | 8.69 | 8.88 | 7.48 | 9.01* |
| Lymphocytes G/l | 2.43 | 2.15 | 2.56 | 2.51 | 3.16 | 2.71 | 2.24 | 2.58 |
| Monocytes, G/l | 0.65 | 0.70 | 0.47 | 0.37 | 1.0 | 0.76* | 0.47 | 0.76 |
| Neutrophils total G/l | 5.91 | 5.11 | 2.70 | 2.64 | 4.13 | 4.9 | 4.46 | 5.63* |
| Neutrophils segm, % | 54.91 | 61.41 | 44.32 | 43.62 | 45.25 | 50.19 | 42.40 | 56.37 |
| Neutrophils stick % | 1.93 | 1.75 | 3.56 | 2.69 | 2.93 | 3.69 | 18 | 4.37 |
| Basophils G/l | 0.050 | 0.053 | 0.037 | 0.049 | 0.049 | 0.037* | 0.065 | 0.076 |
| Eosinophils G/l | 0.29 | 0.18* | 0.20 | 0.24 | 0.14 | 0.18* | 0.13 | 0.15 |
| LUC, % | 3.27 | 4.03* | 4.94 | 5.51 | 3.42 | 3.22 | 3.68 | 1.68* |
| Thrombocytes G/l | 422.9 | 404.3 | 258.6 | 252.7 | 452.1 | 464.4 | 526.5 | 567.1 |

Average of the values at the start of the MemSID trial and the up-dosing phase (base) are compared to the average of the last 3 months of treatments (20 mg/day) and a down-dose phase (end). Stars denote significance ($p<0.05$) between the “base” and the “end” datasets.

**Supplementary figure legends**

**Supplementary Figure 1** Schematic representation of the trial design. Numbers within the arrows indicate number of weeks, number underneath stand for the memantine daily dose.

**Supplementary figure 2.** Example of analysis of the cell abundance per fraction and position of the M fraction. Photographs of the distribution of RBCs on the Percoll density gradient were analyzed using the Plot profile tool of ImageJ software. Color intensity was measured though the whole sample length and plotted graphically. Light, medium and dense fraction borders were determined manually and area under the curve was calculated. Highest color intensity was determined as a position of medium fraction. The obtained value was normalized to the height of the Percoll column (white line) which could vary from image to image.

**Supplementary figure 3.** Details on morphometry of fixed RBCs.

(A) Representative images of fixed RBC from four patients completed the trial obtained via the confocal microscopy. Before = cells fixed at the screening visit, end of treatment = cells fixed

after the last memantine pill was taken by the end of down-dose phase,  post = end of the follow-up period.

(B) Automated and manual analysis of fixed RBC shapes. Fixed RBC shape to projected area distributions based on the manual classification and eccentricity values before and after the treatment and by the end of the follow-up phase. As mentioned in the extended methods section, projected area was defined as number of pixels inside the cell border using Matlab R2017b software routine and re-calculated to $\mu m^2$ thereafter.

(C) Impact of memantine treatment on the solidity and eccentricity of RBCs of individual patients compared to three healthy donors (S1-S3, grey color). Samples were collected at pre-screening (pre, blue color), by the end of down-dosing phase (end, black color) and at the end of the follow-up phase (post, red color). Paired t-tests were used to assess the significance of changes for individual patients with memantine treatment and its interruption.

**Supplementary literature**

Corbett, J.D., Mickols, W.E. & Maestre, M.F. (1995) Effect of hemoglobin concentration on nucleation and polymer formation in sickle red blood cells. *J Biol Chem,* **270,** 2708-2715.

Noetzli, M., Ansermot, N., Dobrinas, M. & Eap, C.B. (2012) Simultaneous determination of antidementia drugs in human plasma: procedure transfer from HPLC-MS to UPLC-MS/MS. *J Pharm Biomed Anal,* **64-65,** 16-25.

Noetzli, M., Choong, E., Ansermot, N. & Eap, C.B. (2011) Simultaneous determination of antidementia drugs in human plasma for therapeutic drug monitoring. *Ther Drug Monit,* **33,** 227-238.

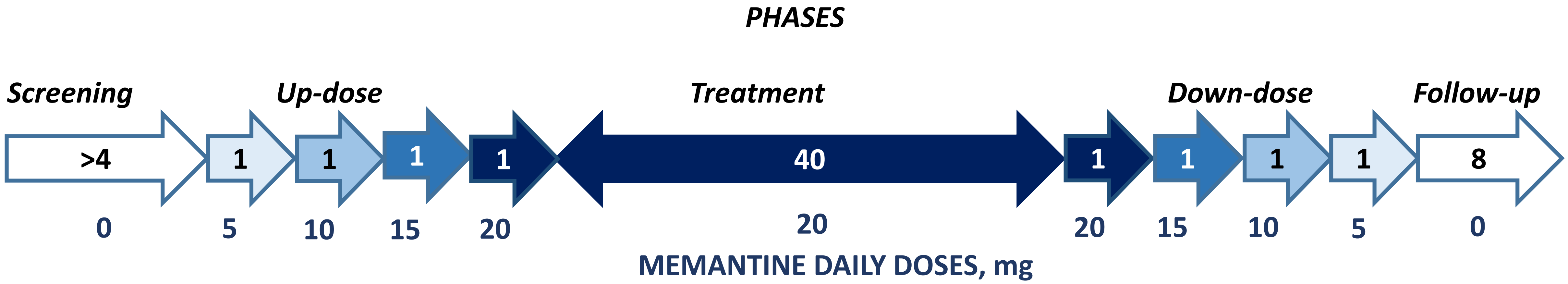
PHASES
Screening
Up-dose
Treatment
Down-dose
Follow-up
>4
1
1
1
1
40
1
1
1
1
8
0
5
10
15
20
20
20
15
10
5
0
MEMANTINE DAILY DOSES, mg

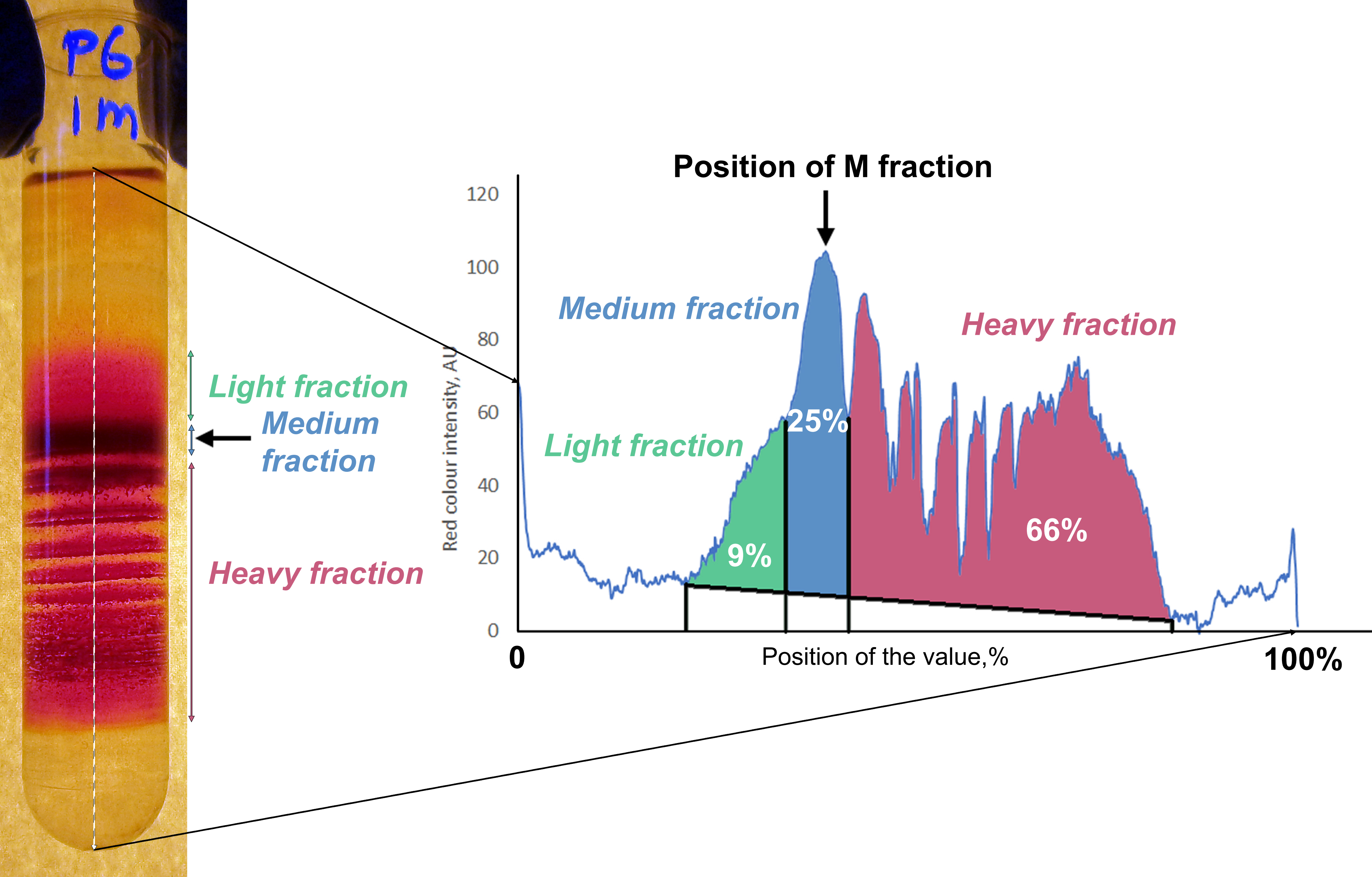

P6
1m
Light fraction
Medium fraction
Heavy fraction
Position of M fraction
Medium fraction
Heavy fraction
Light fraction
25%
9%
66%
Red colour intensity, AU
120
100
80
60
40
20
0
0
Position of the value,%
100%

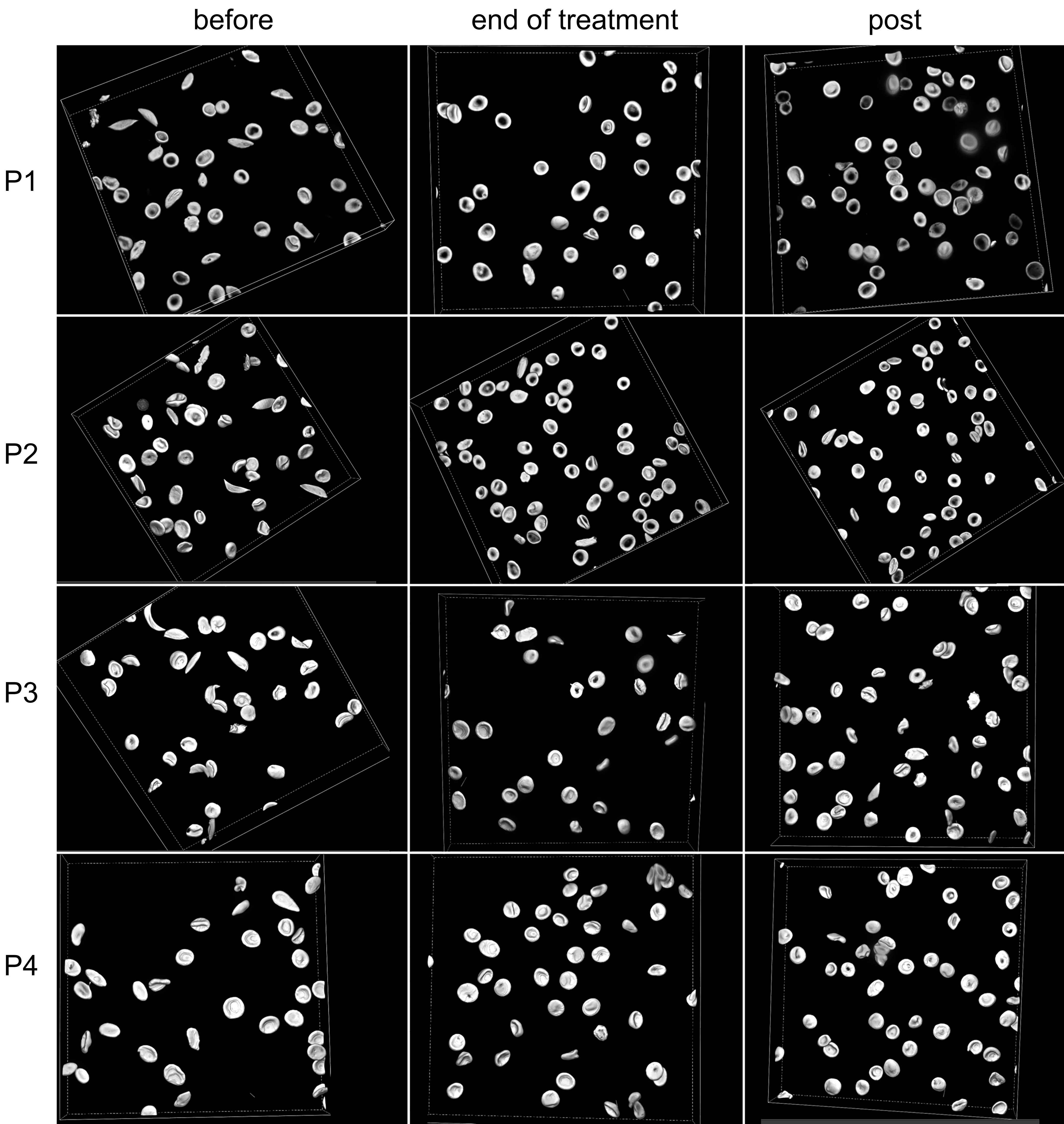
before
end of treatment
post
P1
P2
P3
P4

Relative occurence of classified RBC shapes

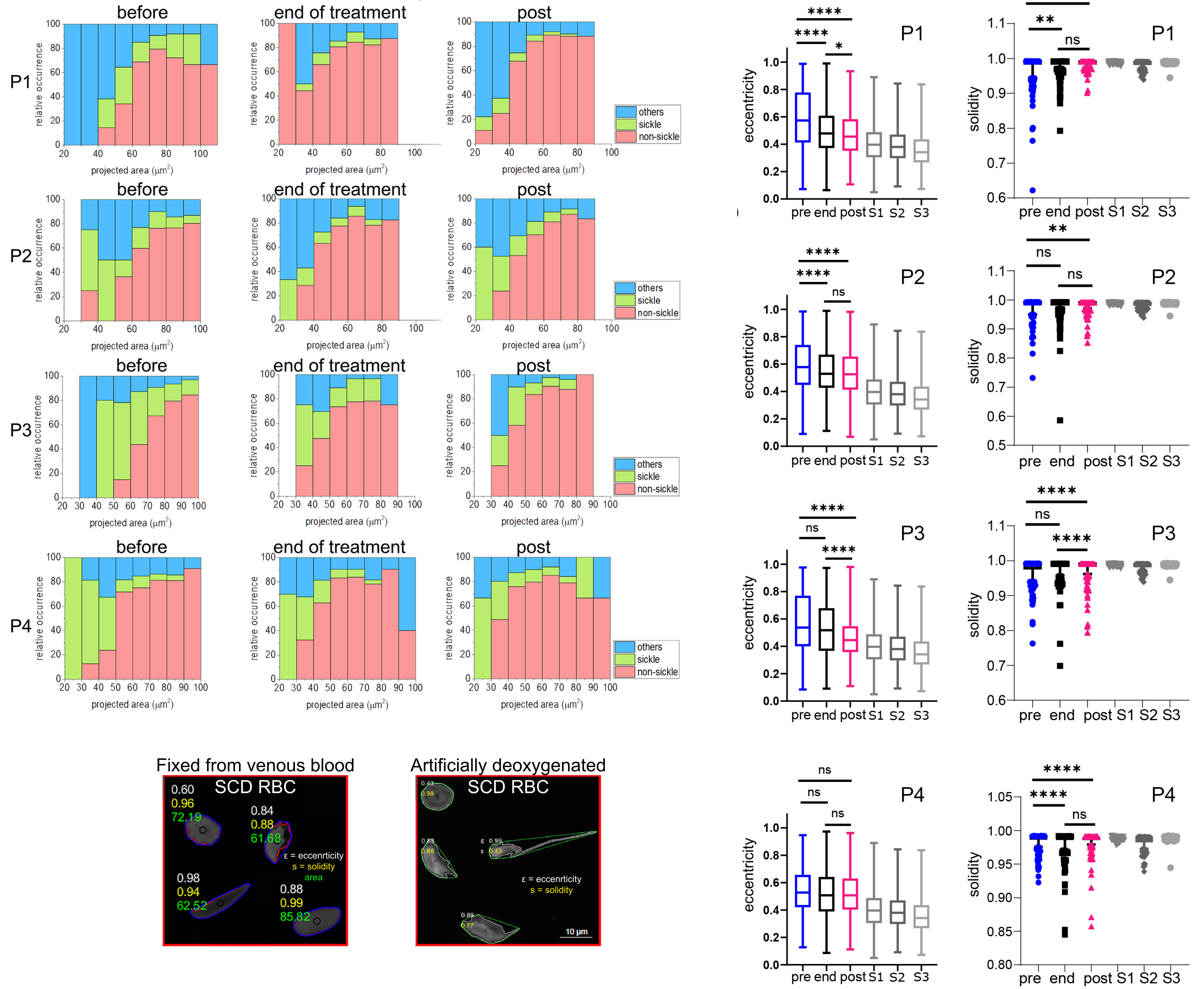

Supplement: Supplementary file 1 — Supporting Information [file JHA2-1-23-s001.pdf]
